# Deep inspiration breath-hold for left-sided breast irradiation: Analysis of dose-mass histograms and the impact of lung expansion

**DOI:** 10.1186/s13014-019-1293-1

**Published:** 2019-06-18

**Authors:** Markus Oechsner, Mathias Düsberg, Kai Joachim Borm, Stephanie Elisabeth Combs, Jan Jakob Wilkens, Marciana Nona Duma

**Affiliations:** 1 Department of Radiation Oncology, Klinikum rechts der Isar, School of Medicine, Technical University of Munich, Ismaninger Str. 22, 81675 München, Germany; 2 Institute of Innovative Radiotherapy (iRT), Helmholtz Zentrum München, Oberschleißheim, Germany; 3 Deutsches Konsortium für Translationale Krebsforschung (DKTK), Partner Site Munich, Munich, Germany; 4 Department of Radiotherapy and Radiation Oncology, Universitätsklinikum Jena, Jena, Germany

**Keywords:** Breast cancer, Deep inspiration breath-hold, Gating, Heart dose, Lung dose, Dose-mass histogram

## Abstract

**Background:**

The aim of this study was to compare dose-volume histogram (DVH) with dose-mass histogram (DMH) parameters for treatment of left-sided breast cancer in deep inspiration breath-hold (DIBH) and free breathing (FB). Additionally, lung expansion and anatomical factors were analyzed and correlated to dose differences.

**Methods:**

For 31 patients 3D conformal radiation therapy plans were retrospectively calculated on FB and DIBH CTs in the treatment planning system. The calculated doses, structures and CT data were transferred into MATLAB and DVHs and DMHs were calculated. Mean doses (Dmean), volumes and masses receiving certain doses (Vx, Mx) were determined for the left lung and the heart. Additionally, expansion of the left lung was evaluated using deformable image registration. Differences in DVH and DMH dose parameters between FB and DIBH were statistically analyzed and correlated to lung expansion and anatomical factors.

**Results:**

DIBH reduced Dmean (DVH) and relative V20 (V20 [%]) of the left lung in all patients, on average by − 19 ± 9% (mean ± standard deviation) and − 24 ± 10%. Dmean (DMH) and M20 [%] were also significantly reduced (− 12 ± 11%, − 16 ± 13%), however 4 patients had higher DMH values in DIBH than in FB. Linear regression showed good correlations between DVH and DMH parameters, e.g. a dosimetric benefit smaller than 8.4% for Dmean (DVH) in DIBH indicated more irradiated lung mass in DIBH than in FB. The mean expansion of the left lung between FB and DIBH was 1.5 ± 2.4 mm (left), 16.0 ± 4.0 mm (anterior) and 12.2 ± 4.6 mm (caudal). No significant correlations were found between expansions and differences in Dmean for the left lung. The heart dose in DIBH was reduced in all patients by 53% (Dmean) and this dosimetric benefit correlated to lung expansion in anterior.

**Conclusions:**

Treatment of left-sided breast cancer in DIBH reduced dose to the heart and in most cases the lung dose, relative irradiated lung volume and lung mass. A mass related dosimetric benefit in DIBH can be achieved as long as the volume related benefit is about ≥8–9%. The lung expansion (breathing pattern) showed no impact on lung dose, but on heart dose. A stronger chest breathing (anterior expansion) for DIBH seems to be more beneficial than abdominal breathing.

## Introduction

Radiation therapy is well-established in the treatment of breast cancer reducing the rate of locoregional recurrence and improving survival rate [1, 2]. However, side-effects have to be considered, which are related to dose to organs at risk (OAR), in particular to the heart and the lung [3–10]. It was shown, that the mean heart dose correlates with the rate of cardiac mortality and coronary events and the dose to the whole lung with the incidence of lung cancer [4–6]. The risk of radiation pneumonitis is also related to the mean lung dose or irradiated lung volume [7–11].

To reduce the risk of short or long term side-effects several techniques are available (e.g. intensity-modulated radiation therapy, treatment in prone position or respiration correlated irradiation) which provide improved dose sparing to the heart and the lung. A promising technique is gated irradiation during deep inspiration breath-hold (DIBH) [12–26]. This technique was investigated by several studies with diverse endpoints, mostly focused on reducing dose to the heart and heart substructures like the left anterior descending coronary artery (LAD) in left sided breast cancer treatment. Compared to treatment in free breathing (FB) mean dose (Dmean) reductions of the heart of 31–63% are achievable in DIBH [16–20, 22, 24–26]. The Dmean of the ipsilateral lung can also be reduced by 7–15% [19, 20, 22, 23, 25]. Further, the relative lung volume receiving a certain dose (Vx) is smaller in DIBH, too. However, the absolute irradiated lung volume increases due to the enlargement of the lung volume.

In contrast to the heart, local density of the lung changes significantly between FB and DIBH. To evaluate the lung dose, the dose-mass histogram (DMH) concept was proposed [27–30] as a more accurate model than the typically used dose-volume histogram (DVH) concept [31]. While the DVH uses volume elements (voxels) which stay unchanged between FB and DIBH, DMH accounts for density changes inside the voxels. Commercial treatment planning systems (TPS) offer no option to calculate DMH. Zurl et al. [23] used a predefined structure in the TPS to determine the lung mass receiving 20 Gy (M20) in FB and DIBH of left-sided breast cancer by multiplying the structure volume with the mean density inside the volume.

The aim of this study was to calculate and compare DMH parameters of the left lung with DVH parameters for treatments of left-sided breast cancer patients in FB and DIBH. Additionally, the lung expansion between FB and DIBH was analyzed with deformable image registration (DIR) to search for possible correlations to dose changes of the left lung and the heart.

## Material and methods

### Patients and image acquisition

A total of 31 patients were retrospectively selected for this study. The patients were treated in our department for left-sided breast cancer between 2013 and 2015. All patients gave their informed consent, both spoken and written before starting radiation therapy, that they will undergo computed tomography (CT) for radiation therapy treatment planning. CT scanning was performed on a Somatom Emotion 16 CT (Siemens Healthineers, Erlangen, Germany). The patients were positioned on a wing board with the arms above their head. A 15 to 30 min training was performed with all patients where they were instructed to perform chest breathing for the DIBH. For monitoring of the patients’ breathing curves the real-time position management system (RPM, Varian Medical Systems, Palo Alto, CA) was used and individual gating windows were defined depending on the depth of inspiration. Two scans were performed in each case, both with a slice thickness of 3 mm. The first scan was a slow planning CT during FB and the second was acquired during DIBH.

### Treatment planning and evaluation

Contouring and treatment planning were performed with the TPS Eclipse 13.0 (Varian Medical Systems, Palo Alto, CA, USA). Both was done retrospectively for this study by a single senior radiation oncologist and an experienced medical physicist to allow a fair comparison between FB and DIBH. Planning target volumes (PTV) and organs at risk were contoured on FB and DIBH CT datasets according to the Radiation Therapy Oncology Group contouring atlas. All treatment plans were calculated on both CT datasets for every patient. A 3-dimensional conformal radiation therapy (3D-CRT) technique was used, consisting of 2 opposing tangential beams and additional beam segments (1–5). The latter were applied to improve target dose coverage and homogeneity. Dose was calculated with the anisotropic analytical algorithm (AAA) and a grid size of 2.5 × 2.5 mm^2^. The prescription dose to the PTV was 50 Gy delivered in 2 Gy per fraction and the plans were normalized to a median PTV dose corresponding to the prescription dose.

All calculated doses, the CT data and the contoured structures were transferred to the software MATLAB (The MathWorks, Natick, MA, USA). DVHs and DMHs for the left lung and the heart were calculated in MATLAB using self-written programs. For DMH calculation the Hounsfield unit (HU) to electron density conversion table of the TPS was used to assign mass density values to the voxels of the CT datasets.

The following DVH- and DMH-parameters for the left lung were determined for FB and DIBH plans: Mean dose (Dmean), volumes (Vx) and masses (Mx) receiving at least a certain dose. Vx and Mx of the left lung were determined as relative [%] and absolute values [cm^3^, g]. Dmean, V20 [%] and V40 [%] from DVH were also evaluated for the heart. Furthermore, the following anatomical factors were calculated: PTV volume and lung volume of the ipsilateral lung in FB and DIBH, change in lung volume between DIBH and FB (∆ lung volume), ipsilateral lung mass and density. Differences between values in DIBH and FB were always calculated as “value (DIBH) - value (FB)” and were denoted with “∆”, e.g. ∆Dmean.

### Evaluation of lung expansion

The lung expansion between FB and DIBH was analyzed using deformable image registration. For this purpose an automated workflow was implemented in MATLAB, consisting of three steps. In a first step the FB and the DIBH CT datasets were separated into lung and non-lung tissue, using the structure sets from the TPS. In a second step, a non-rigid image registration between the separated datasets was performed. For this step the B-Spline algorithm from the open source image registration framework Plastimatch (www.plastimatch.org) was utilized, which optimized the deformation over 6 stages using the mean squared error metric. The deformation vector fields (DVFs) of the lung and the non-lung tissue were merged in the third step and then applied to the contoured structures. The result of the image deformation were visually inspected by overlaying the CT dataset and the DVF. For that purpose we used the open source software 3D Slicer (www.slicer.org). To evaluate the expansion of the left lung and the lung V20 in left, anterior and caudal direction, the mean values of the DVF within these structures were calculated. Additionally, the 3D expansion was calculated from the mean expansions in all three directions.

### Statistics

Statistical analysis was performed using SPSS Version 25.0 (SPSS Inc., Chicago, IL, USA). Dose in FB and DIBH were compared for statistically significant differences using the Wilcoxon-Test. Linear regression analysis was applied to analyze differences between DMH and DVH parameters. Correlations between anatomical factors and dose differences were determined by Spearman’s rank correlation coefficient (r). A *p*-value < 0.05 indicates statistically significant differences.

## Results

### Characteristics and dose to the left lung and the heart

The mean left lung volume of all 31 patients was 1432 ± 290 cm^3^ (mean ± standard deviation (SD)) in FB und 2581 ± 321 cm^3^ in DIBH. The total lung mass showed a good accordance between FB and DIBH, whereas the mean lung density decreased from 0.31 ± 0.05 g/cm^3^ (FB) to 0.17 ± 0.03 g/cm^3^ (DIBH) (Table 1).

**Table 1:** PTV volume, anatomical characteristics and dose parameters of the left lung and the heart in FB and DIBH and the differences (∆)

	FB	DIBH	∆ (DIBH-FB)	∆ (DIBH-FB)/FB
	mean ± SD	mean ± SD	mean ± SD	mean ± SD
PTV volume [cm^3^]	969 ± 377	956 ± 374	− 13 ± 32	− 1 ± 3%
left lung				
volume [cm^3^]	1432 ± 290	2581 ± 321	1149 ± 261	84 ± 26%
mass [g]	439 ± 68	443 ± 76	4 ± 46	1 ± 10%
density [g/cm^3^]	0.31 ± 0.05	0.17 ± 0.03	−0.14 ± 0.04	− 44 ± 7%
Dmean (DVH) [Gy]	10.0 ± 1.7	8.1 ± 1.6	− 1.9 ± 1.0	−19 ± 9%
Dmean (DMH) [Gy]	8.3 ± 1.5	7.3 ± 1.5	−1.0 ± 0.9	−12 ± 11%
V20 [%]	18.9 ± 3.6	14.4 ± 3.3	− 4.5 ± 2.1	− 24 ± 10%
V20 [cm^3^]	267.5 ± 67.0	367.2 ± 86.6	99.7 ± 69.4	40 ± 29%
M20 [%]	15.0 ± 3.0	12.6 ± 2.9	− 2.4 ± 1.9	− 16 ± 13%
M20 [g]	65.3 ± 16.0	55.7 ± 16.4	− 9.6 ± 11.7	− 14 ± 18%
heart				
Dmean (DVH) [Gy]	4.0 ± 1.9	1.7 ± 1.0	− 2.3 ± 1.4	− 53 ± 19%
V20 [%]	6.2 ± 4.2	1.2 ± 1.0	− 5.0 ± 3.1	−83 ± 23%
V40 [%]	3.6 ± 2.7	0.4 ± 0.9	−3.3 ± 2.2	−87 ± 26%

*PTV* planning target volume, *DVH* dose-volume histogram, *DMH* dose-mass histogram, *FB* free breathing, *DIBH* deep inspiration breath-hold, *SD* standard deviation

Irradiation in DIBH resulted in a significant reduction of Dmean to the left lung of the DVH from 10.0 ± 1.7 Gy (FB) to 8.1 ± 1.6 Gy (DIBH) (*p* < 0.01, Table 1). DIBH reduced the Dmean (DVH) in all patients. Dmean of the DMH decreased from 8.3 ± 1.5 Gy (FB) to 7.3 ± 1.5 Gy (DIBH) (p < 0.01) and decreased in 27 patients and increased in 4 patients.

**Fig. 1:**
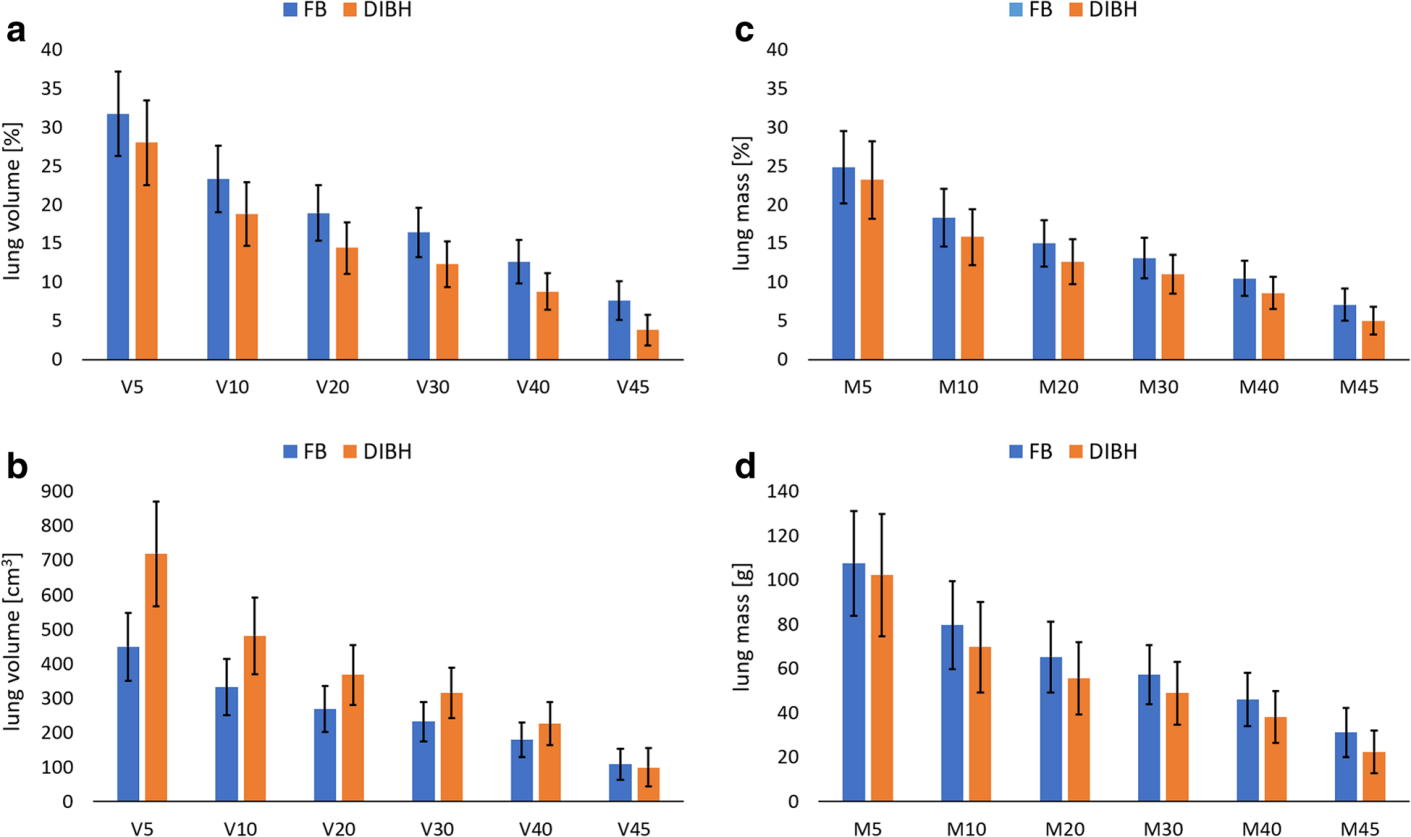
Mean volumes (V5–45, **a**, **b**) and masses (M5–45, **c**, **d**) of the left lung receiving a certain dose, presented as relative and absolute values ± SD. *FB* free breathing, *DIBH* deep inspiration breath-hold

**Fig. 2:**
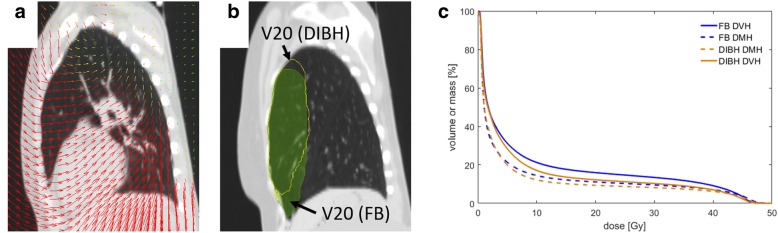
Representation of the deformation vector field of a patient (**a**) on a sagittal slice in DIBH. The arrows show the direction of expansion between DIBH and FB. The difference between the V20 contours of the left lung in FB and DIBH is presented in (**b**). The green area shows V20 in FB and the yellow outline V20 in DIBH, which was deformed to the FB CT. As can be seen there are differences in the location of irradiated lung volumes. A comparison of DVH and MDH of the left lung for a patient is plotted in (**c**)

Figure 1 compares irradiated lung volumes (V5–45) and lung mass (M5–45) in FB and DIBH, calculated as relative [%] and absolute values [cm^3^, g], respectively. V5–45 [%] were always smaller in DIBH than in FB, whereas V5–45 [cm^3^] were larger in DIBH, except for V45. For the irradiated lung mass the means of M5–45 were always smaller in DIBH than in FB. However, a few patients had an increased irradiated lung mass in DIBH (e.g. 4 patients for M20) or a decreased absolute irradiated lung volume in DIBH (e.g. 3 for V20). A comparison between the DVH and DMH of a patient is presented in Fig. 2c.

The mean dose to the heart was reduced in DIBH for all patients and decreased significantly from 4.0 ± 1.9 Gy (FB) to 1.7 ± 1.0 Gy (DIBH) (*p* < 0.01). Volume reduction of V20 [%] and V40 [%] was − 83% and − 87% (Table 1).

**Fig. 3:**
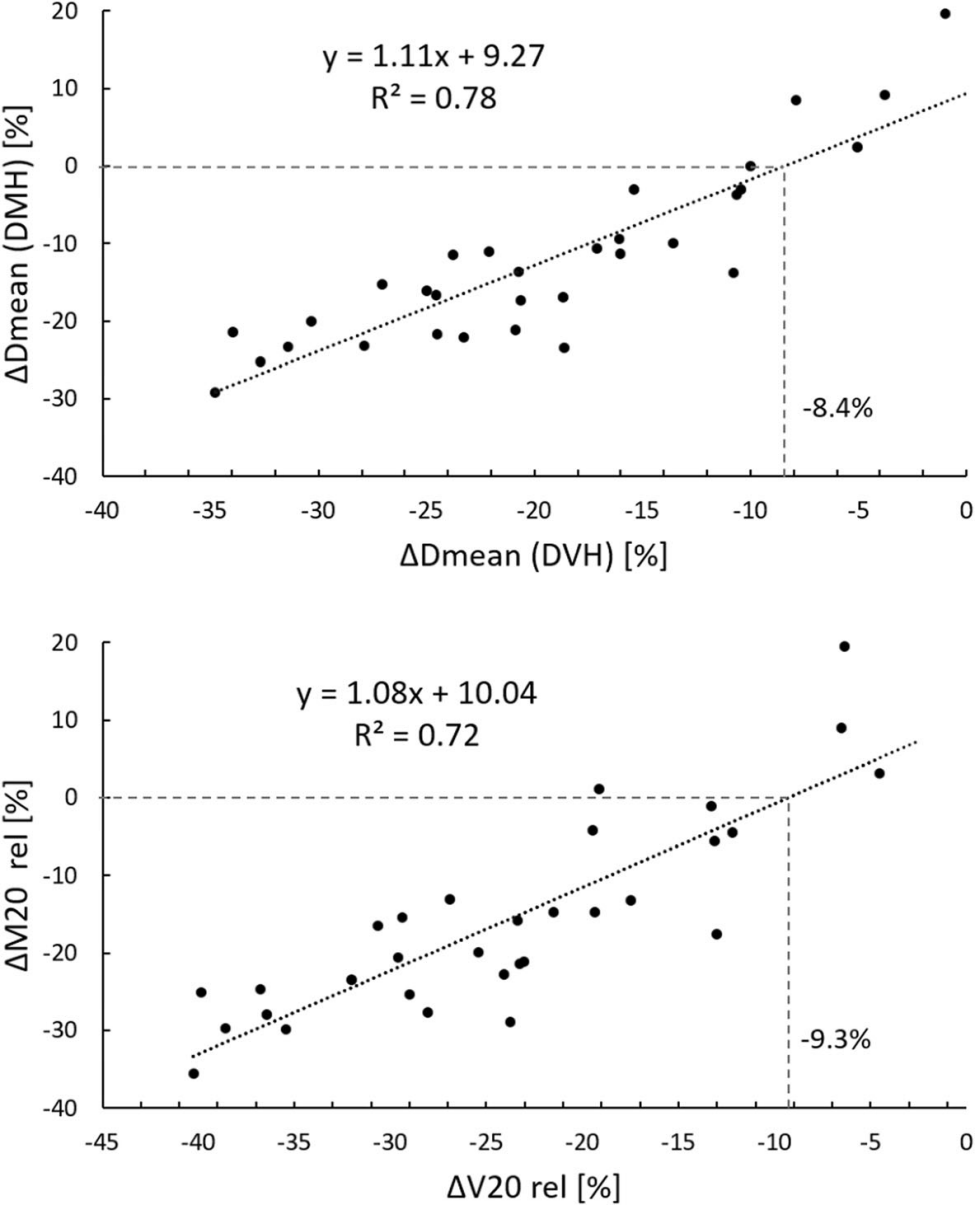
Regression analysis showing the correlation between lung dose parameters from DVH and DMH. The dashed lines mark the DVH values corresponding to a DMH value equal to zero. *y* slope of the line, *R*^*2*^ coefficient of determination.

Regression analysis was used to evaluate the correlations between DVH and DMH parameters of the left lung. Figure 3 shows the correlations for ∆Dmean (DVH vs. DMH) and ∆V20 vs. ∆M20 [%]. Good correlations were achieved between the DVH and DMH parameters. From the linear regression it can be estimated, that DIBH led to a reduction in Dmean (DMH) if Dmean (DVH) was reduced by at least − 8.4%. A benefit in irradiated lung mass in DIBH for ∆M20 [%] was achieved for ∆V20 [%] ≤ − 9.3%.

### Expansion of the left lung and V20

**Fig. 4:**
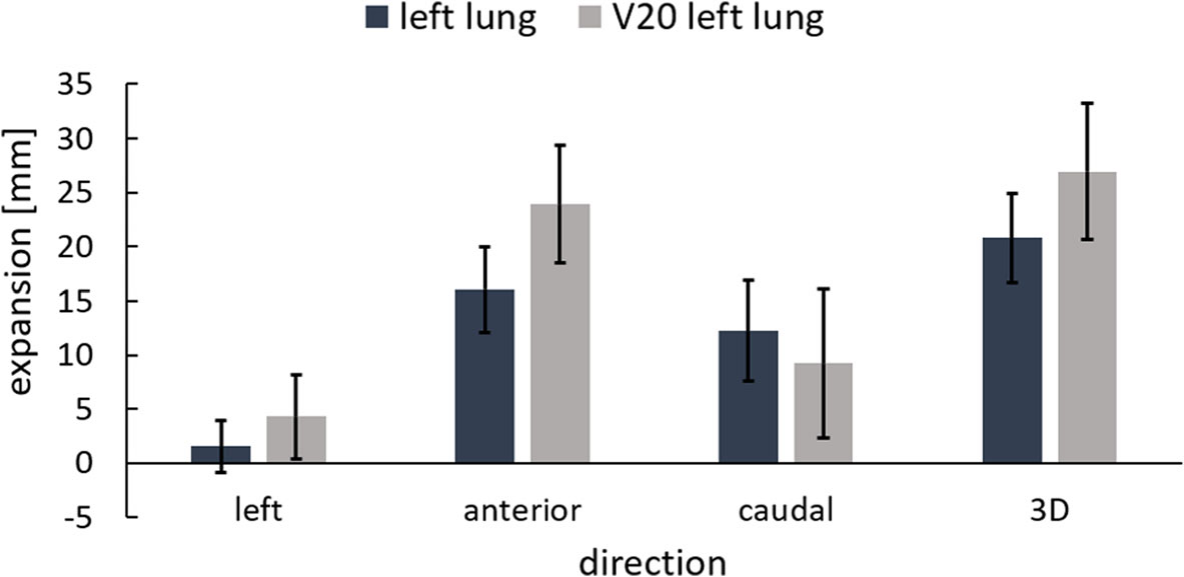
Mean expansion ± SD over all patients between FB and DIBH for the left lung and V20 in left, anterior and caudal direction and the 3D expansion

To analyze the lung expansion between FB and DIBH deformation vector fields were calculated with DIR and evaluated. Figure 2a shows an example of the DVF of a patient. The mean expansion of the left lung between FB and DIBH over all patients was 1.5 ± 2.4 mm to the left side of the patients, 16.0 ± 4.0 mm in anterior and 12.2 ± 4.6 mm in caudal direction. The 3D expansion was 20.8 ± 4.1 mm. The lung volume V20 showed larger expansions of 4.3 ± 3.9 mm to the left, 23.9 ± 5.5 mm in anterior and 9.2 ± 6.9 mm in caudal direction with a 3D expansion of 27.0 ± 6.3 mm (Fig. 4).

Further we analyzed the lung regions in FB and DIBH which were irradiated with at least 20 Gy (V20). For this purpose we qualitatively compared the volume V20 in FB with the volume V20 in DIBH which was deformed from the DIBH-CT to the FB-CT by the deformable image registration. The main difference between both volumes existed in the cranio-caudal direction (Fig. 2b). V20 in FB was more caudal located and was shifted towards cranial in DIBH. This effect could be seen in all patients in our collective.

Furthermore, we calculated the difference between the expansion of the left lung in caudal and anterior, normalized to the anterior expansion and expressed as percentage. A negative value means a larger expansion in anterior than in caudal direction, corresponding to a stronger chest breathing for DIBH. A positive value stands for larger expansion in caudal direction which points to a stronger abdominal breathing. The mean difference over all patients was − 18.8 ± 37.4% with a range between − 89.4 and 54.0%. Overall 22 patients had a larger expansion in anterior and 9 patients in caudal direction.

**Fig. 5:**
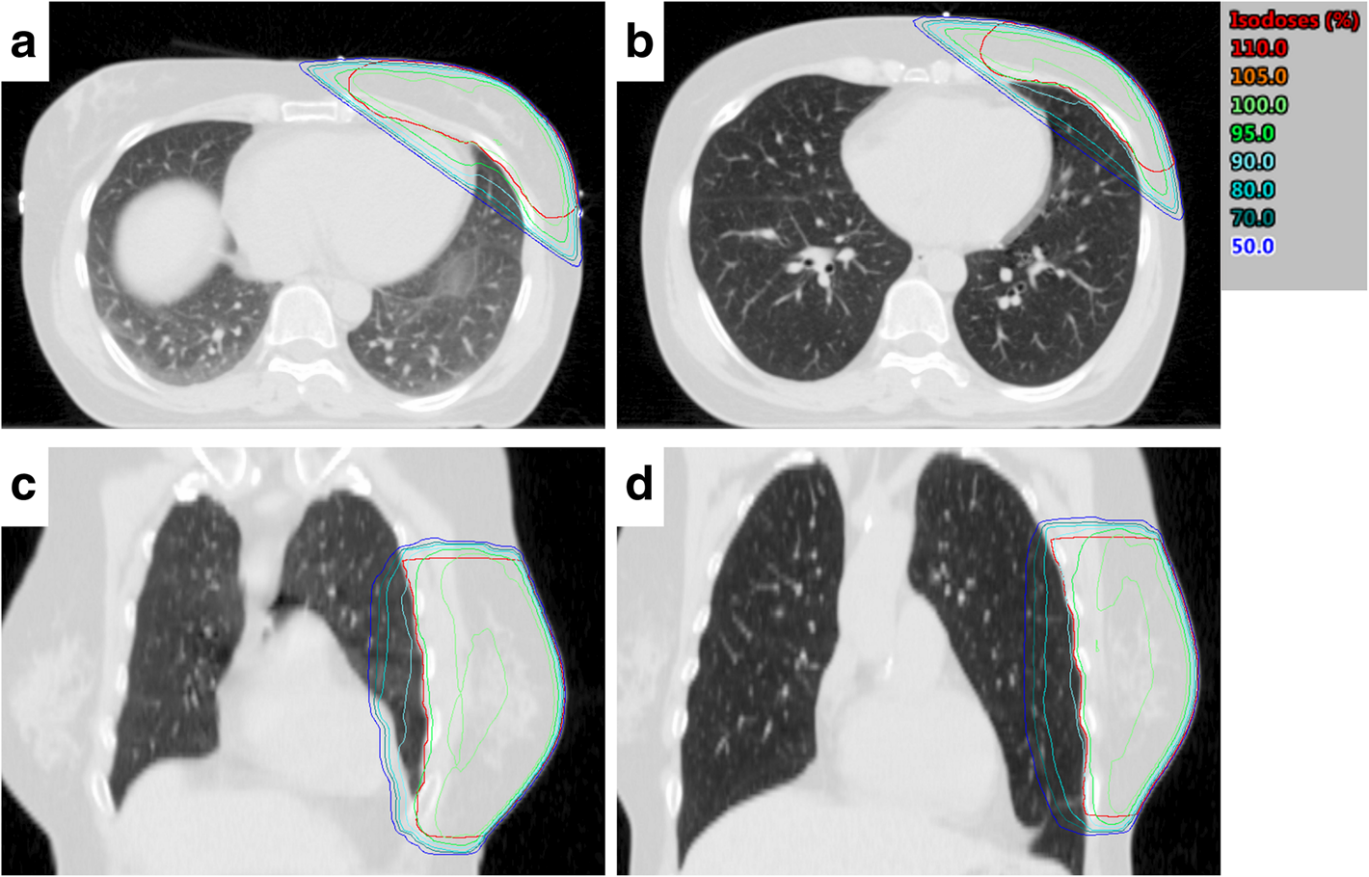
Two patient cases, which had higher irradiated lung mass in DIBH than in FB. For the first patient a large amount of heart tissue inside the treatment field (**a**) is replaced by lung tissue (**b**). For the second patient the PTV in FB extends over the abdomen (**c**) and is shifted towards the lung in DIBH (**d**)

In 4 cases the irradiated lung mass for M20 was higher in DIBH than in FB. Taking a closer look at these patients, we found, that in 3 cases a large amount of heart tissue irradiated in FB was replaced by lung tissue, which increased the irradiated lung mass in DIBH (Fig. 5a and b). In one case the PTV in FB extended beyond the left lung to the abdomen. In DIBH the abdominal tissue was replaced by lung tissue increasing the irradiated lung mass (Fig. 5c and d).

### Correlations

Dose differences between DIBH and FB for the lung and the heart were analyzed for correlations to anatomical factors. The differences in dose parameters were calculated as differences between DIBH and FB, normalized to the FB values and expressed as percentage, e.g. ∆V20 relative [%] = (V20_DIBH_ [%] - V20_FB_ [%])*100 / V20_FB_ [%] or ∆Dmean lung [%] = (Dmean lung _DIBH_ - Dmean lung _FB_)*100 / Dmean lung _FB_. Spearman’s correlation coefficients were calculated and are summarized in Table 2. Because differences (∆) were calculated as value(DIBH) - value (FB) a negative correlation coefficient means a benefit for DIBH compared to FB.

**Table 2:** Spearman’s correlation coefficient between differences in dose parameters (DIBH-FB) and anatomical factors or expansions of the left lung

	PTV volume in FB [cm^3^]	lung volume in FB [cm^3^]	Δlung volume [%]	left-expansion	anterior-expansion	caudal-expansion	3D expansion
ΔDmean lung (DVH) [%]	**0.37** ^*^	−0.12	0.13	0.18	−0.02	0.00	0.07
ΔDmean lung (DMH) [%]	**0.45** ^*^	0.00	0.22	0.19	0.10	0.03	0.17
ΔV20 relative [%]	**0.40** ^*^	−0.11	0.11	0.17	−0.03	− 0.04	0.04
ΔM20 relative [%]	**0.45** ^*^	0.00	0.24	0.21	0.14	−0.04	0.18
ΔDmean heart [%]	−0.27	**0.44** ^*^	**−0.47** ^**^	− 0.30	**−0.51** ^**^	− 0.10	−0.35

Differences (∆) in dose parameters were calculated as relative values, e.g. ∆V20 relative [%] = (V20_DIBH_ [%] - V20_FB_ [%])*100 / V20_FB_ [%] or ∆Dmean lung [%] = (Dmean lung _DIBH_ - Dmean lung _FB_)*100 / Dmean lung _FB_. Because differences (∆) were calculated as value(DIBH) - value(FB) a negative correlation coefficient means a benefit for DIBH compared to FB

*PTV* planning target volume, *DVH* dose-volume histogram, *DMH* dose-mass histogram, *FB* free breathing

*significant correlations with *p* < 0.05, ** significant correlations with *p* < 0.01

The PTV volume in FB correlated significantly to differences in dose parameters of the left lung showing smaller benefits in DIBH with larger PTV volumes. PTV volume in DIBH was quite similar to PTV volume in FB (Tab 1) and resulted in nearly the same correlations (data not shown).

A larger lung volume in FB decreased the dose sparing benefit of DIBH for the heart. Larger ∆lung volume [%] (deeper inspiration) correlated to higher differences in Dmean of the heart between DIBH and FB. Expansions of the left lung to the left, anterior, caudal and the 3D expansion showed no significant correlations to differences in lung dose parameters. The reduction in heart dose (∆Dmean) in DIBH correlated with lung expansion in anterior, but not to caudal expansion.

## Discussion

This study showed that treatment of left-sided breast cancer in DIBH reduced, besides the DVH-based heart and lung dose, also the irradiated lung mass. Despite an increase in absolute irradiated left lung volume in DIBH, the irradiated lung mass was reduced in 87% of our patients. Dose reductions of Dmean (DVH) and V20 of 7–15% [19, 20, 22, 23, 25] and 9–18% [16, 18, 19, 22–25] are reported in the literature. The dose reductions in DIBH for the left lung in our study were somewhat higher (∆Dmean = − 19%, ∆V20 = − 24%). However, there is a large variability in reported lung dose reductions and a study by Walston et al. reported even no significant dose reduction for V20 and Dmean [26].

Due to lung expansion in DIBH significantly larger absolute lung volumes are irradiated as compared to FB, whereas the relative irradiated volume is reduced. For calculation of the DVH the lung is divided into equal volume elements (voxels). However, unlike e.g. the heart, there is a change in lung density inside the voxels in the lung between FB and DIBH. The mass density inside the voxels is reduced, which is not taken into account in DVH calculation. Butler et al. [28] proposed the DMH concept taking such changes in tissue density into account. Today, calculation of DMH is not routinely implemented in available TPSs. Zurl et al. [23] analyzed irradiated lung mass between FB and DIBH in a commercial TPS using predefined structures. By multiplying the volume of the V20 with the mean density inside this structure they calculated M20.

In our work, we used self-written programs in MATLAB to calculate the whole DMH and Dmean (DMH) for the ipsilateral lung. In total, DIBH reduced Dmean (DMH) by 12% and the irradiated lung mass, e.g. M20 by 16%, which is very close to the 17% reduction in M20 reported in [23]. In contrast to Dmean (DVH) and V20, where lung dose of all patients was reduced, 4 patients had an increased Dmean (DMH) and M20 in DIBH. Dmean (DVH) was higher than Dmean (DMH), which was also reported by Fogliata et al. [32]. We found a good linear correlation between these parameters and also between V20 and M20. From our data we can estimate, that the mass related dosimetric benefit of DIBH for the left lung disappears, if there is no significant reduction of the Dmean (DVH) to the left lung in DIBH. If Dmean (DVH) is solely 8% lower (9% for V20) in DIBH than in FB, no benefit in lung sparing can be expected (Fig. 3).

The increased irradiated lung mass, which was found in four patients, can be explained by special anatomical situations (Fig. 5) and might be of interest for the decision if irradiation should be performed in FB or in DIBH.

The differences between DVH and DMH impacts also on calculation of complication probabilities using radiobiological models, which was demonstrated by Mavroidis et al. [30]. However, it has to be mentioned, that the theoretical advantages to DMH over DVH has yet not been proven for patients. Clinical data are all based on DVH parameters and further studies are warranted to validate the benefit of DMH for patients.

It has to be noted that dose calculation in low density tissues like the lung depends on the applied dose calculation algorithm. The AAA algorithm was used in this work, which is a limitation of this study. For lung tissue, i.e. areas with high gradients in electron density, modern Monte Carlo based algorithms or the Acuros XB from Varian achieve higher accuracy [32–36]. AAA tends to overestimate the dose in these regions [35, 36]. Fogliata et al. [32] calculated differences of 2% in FB and 4% in DIBH between AAA and a Monte Carlo based algorithm. Furthermore, DMH calculations are stronger affected by uncertainties than DVH calculations. Such uncertainties can arise from HU estimation in low density tissue like the lung, the conversion between HU and mass density or variability in contouring and segmentation. Those limitations were already discussed in Ref [23].

Nonetheless, the reduction of dose to the heart and heart substructures is usually the main objective for using irradiation in DIBH. It was shown, that DIBH can decrease Dmean to the heart by 31–63% [16–20, 22, 24–26]. The results of our study showed a reduced mean heart dose of 53%. The heart dose differences between FB and DIBH are caused by the expansion of the lung, which shifts the heart away from the treatment field. However, the relationship between lung expansion and dose reduction to the heart is not yet completely understood.

Another aim of this work was therefore to analyze this lung expansion into the three dimensions. The lung expansion between FB and DIBH was evaluated by using the deformation vector fields of DIR. In a previous work, DIR and the resulting DVFs have been used to evaluate movement of the breast cavity during free respiration [37]. Applying the DVFs to analyze the expansion of the left lung between FB and DIBH, there was only a small expansion to the patients’ left side and large expansions in anterior and caudal direction. Before CT acquisition the patients were instructed to perform chest breathing for DIBH with the recommendation to reduce abdominal breathing as much as possible. Nevertheless, lung expansion between FB and DIBH is very patient specific. In 22 patients anterior expansion was larger than caudal expansion and in 9 patients it was reverse.

The lung expansions showed no significant correlations to reductions in lung dose parameters (Table 2). In contrast to that, the reduction in heart Dmean correlated significantly to the anterior expansion of the left lung as well as to the difference in lung volume. However, caudal expansion did not. Thus, to reduce the dose to the heart it seems to be beneficial for the patient to be instructed to perform chest breathing for DIBH.

Furthermore, the expansion of the V20 between FB and DIBH was analyzed. As can be expected, expansions of V20 were larger than expansions of the whole left lung with distinctly larger expansions in anterior than in caudal direction (Fig. 4). In DIBH the PTV volume is shifted, mainly in anterior and cranial direction [38]. In accordance to this PTV shift the irradiated lung areas differ between FB and DIBH with more caudal irradiated parts in FB and more cranial parts in DIBH (Fig. 2b).

In addition to lung expansion, further anatomical factors were found to correlate to differences in dose parameters between DIBH and FB. Especially the PTV volume, corresponding to the breast size, had a negative impact on dose sparing for the left lung in DIBH. Dose reductions of heart Dmean in DIBH were smaller for larger lung volumes and increased with larger ∆lung volumes or lung expansions in anterior. A study of Tanguturi et al. [26] found that younger patient age, higher body mass index (BMI) and larger ∆lung volume correlated to a reduced heart dose in DIBH. Czeremszynska et al. [25] achieved positive correlations between BMI, cardiac contact distance or PTV size and dose sparing of the heart and with larger lung volumes in FB having a negative effect, which is in accordance to our findings. Besides these anatomical factors, patients with a favorable tumor prognosis, high mean heart dose or high baseline risk for ischemic heart were identified to have the highest benefit from treatment in DIBH [39]. The authors stated, that the DIBH technique should ideally be offered to all patients with left-sided breast cancer. All patients in our collective benefitted from treatment in DIBH related to heart and lung dose from DVH. In most patients dose to the left lung mass could be reduced too. From this point of view it would be helpful to find factors to identify patients who will not benefit from treatments in DIBH, e.g. due to increased lung dose or if an intensity modulated technique might be better.

## Conclusion

Treatment oft left-sided breast cancer in DIBH reduces heart dose and also lung dose in most patients. Despite an increased absolute irradiated lung volume the relative lung volume and the relative and absolute irradiated lung mass is reduced in most cases. A mass related dosimetric benefit of DIBH for the left lung can be expected, if Dmean (DVH) is at least 8% lower (9% for V20 [%]) in DIBH than in FB. The lung volume and expansion showed no significant correlations to reductions in lung dose. However, lung expansion in anterior correlated to reduction in heart dose, which indicates, that the breathing pattern might be relevant for heart sparing, i.e. chest breathing for DIBH seems to be more beneficial than abdominal breathing.

## Abbreviations

3D: 3-dimensional
3D-CRT: 3-dimensional conformal radiation therapy
AAA: anisotropic analytical algorithm
BMI: body mass index
CT: computed tomography
DIBH: deep inspiration breath-hold
DIR: deformable image registration
Dmean: mean dose
DMH: dose-mass histogram
DVF: deformation vector field
DVH: dose-volume histogram
FB: free breathing
Gy: Gray
HU: Hounsfield unit
LAD: left anterior descending coronary artery
Mx: mass receiving a certain dose
OAR: organs at risk
PTV: planning target volume
TPS: treatment planning system
Vx: volume receiving a certain dose
